# A novel technique for endoscopic stepwise clamping and resection of giant pedunculated colonic polyps

**DOI:** 10.1055/a-2744-0110

**Published:** 2025-12-08

**Authors:** Muqing Wang, Wen Wang, Jianhong Sun, Yanjuan Lin, Yaokui Huang

**Affiliations:** 1 Department of Endoscopy Center 499791 Shantou Central HospitalShantouChina; 2 Department of Gastroenterology, The 900th Hospital of People’s Liberation ArmyFuzhou General Clinical Medical College, Fujian Medical UniversityFuzhouChina; 3 Department of Pathology 499791 Shantou Central HospitalShantouChina

For giant pedunculated colonic polyps, conventional snare polypectomy may not achieve complete en bloc resection and carries a risk of severe bleeding 1 . Although endoscopic submucosal dissection allows for pre-treatment of the stalk vessels, the process of submucosal dissection and wound closure is often challenging due to poor visibility and restricted operability caused by the massive polyp head occupying the colon lumen 2 3 . We have developed a feasible novel technique that effectively enables the stepwise clamping and resection of the stalk in such cases ( Fig. 1 and Video 1 ).

**Fig. 1:**
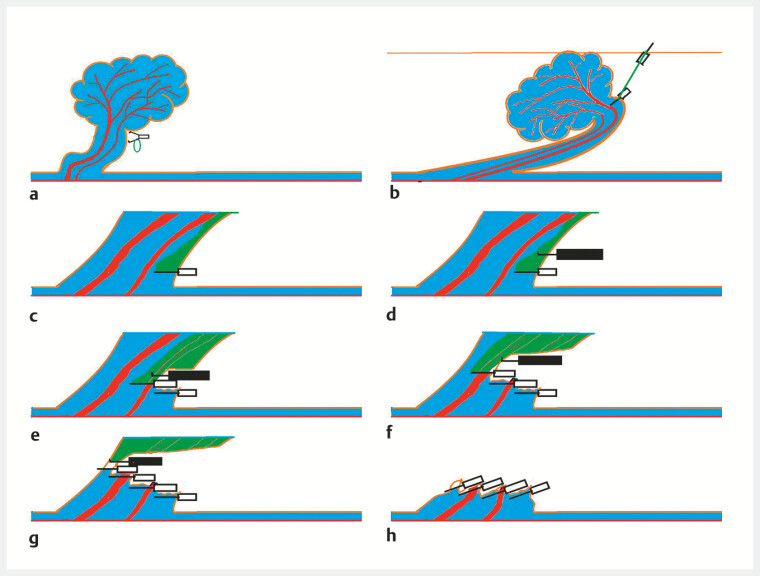
Schematic representation of the novel technique. **a** A clip with an orthodontic rubber band attached to the stalk. **b** The polyp fixed with another clip to the anal side of the intestinal tract. **c** Clips used to clamp the stalk. **d** Cut the stalk above the level of the clips with a hook knife. **e–g** The procedure – clipping followed by cutting – was repeated stepwise. **h** The defect was closed with clips.

A novel technique for endoscopic stepwise clamping and resection of giant pedunculated colonic polyps.Video 1

A 24-year-old man presented with Juvenile Polyposis-Hereditary Haemorrhagic Telangiectasia Overlap Syndrome due to congenital SMAD4 deficiency. Colonoscopy revealed a giant pedunculated polyp with a 45-mm head obstructing the sigmoid colon lumen. We tried to enclose it directly with a snare, but failed. Subsequently, we first applied the double-clip traction with an orthodontic rubber band attached to the stalk, offering excellent visualization and tension. Next, clips were used to clamp the polyp stalk to occlude the feeding vessels. A hook knife was then employed to cut the stalk above the level of the clips. Minor bleeding occurred during the cutting process, but hemostasis was achieved promptly and effectively by electrocoagulation. This procedure – clipping followed by cutting – was repeated stepwise until the lesion was completely resected. Finally, we confirmed that the clips provided secure closure of the defect. The patient was discharged successfully on the third postoperative day, and follow-up revealed no complications, such as delayed bleeding. Histological analysis confirmed the polyp as a villous adenoma with complete resection ( Fig. 2 ).

**Fig. 2:**
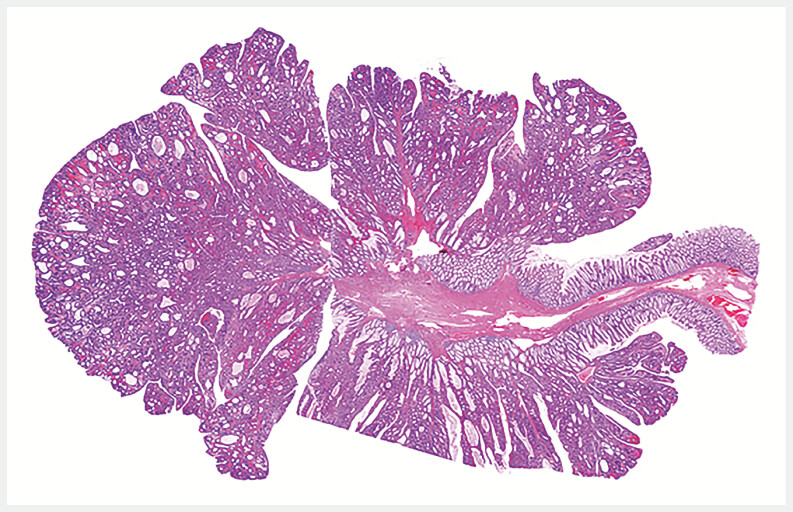
Histopathology showed the polyp as a villous adenoma with complete resection (H & E stain, 5×).

This novel technique of stepwise clamping and resection is suitable for pedunculated lesions. As the technique accomplishes both resection and secure wound closure simultaneously, it merits clinical promotion, and prospective studies are warranted to gather much more reliable evidence.

Endoscopy_UCTN_Code_TTT_1AQ_2AD_3AD

## Notes

Endoscopy E-Videos https://eref.thieme.de/e-videos
*E-Videos* is an open access online section of the journal *Endoscopy* , reporting on interesting cases and new techniques in gastroenterological endoscopy. All papers include a high-quality video and are published with a Creative Commons CC-BY license. Endoscopy E-Videos qualify for HINARI discounts and waivers and eligibility is automatically checked during the submission process. We grant 100% waivers to articles whose corresponding authors are based in Group A countries and 50% waivers to those who are based in Group B countries as classified by Research4Life (see: https://www.research4life.org/access/eligibility/ ). This section has its own submission website at https://mc.manuscriptcentral.com/e-videos .
