# Correction: A novel technique for endoscopic stepwise clamping and resection of giant pedunculated colonic polyps

**DOI:** 10.1055/a-2904-1976

**Published:** 2026-07-01

**Authors:** Muqing Wang, Wen Wang, Jianhong Sun, Yanjuan Lin, Yaokui Huang

**Affiliations:** 1 Department of Endoscopy Center 499791 Shantou Central HospitalShantouChina; 2 Department of Gastroenterology, The 900th Hospital of People’s Liberation ArmyFuzhou General Clinical Medical College, Fujian Medical UniversityFuzhouChina; 3 Department of Pathology 499791 Shantou Central HospitalShantouChina



In the above-mentioned article the affilation for the author Wen Wang has been corrected. This was corrected in the online version on June 30, 2026.

